# Effects of Future Information and Trajectory Complexity on Kinematic Signal and Muscle Activation during Visual-Motor Tracking

**DOI:** 10.3390/e23010111

**Published:** 2021-01-15

**Authors:** Linchuan Deng, Jie Luo, Yueling Lyu, Rong Song

**Affiliations:** 1 Key Laboratory of Sensing Technology and Biomedical Instrument of Guangdong Province, Sun Yat-Sen University, Guangzhou 510006, China; denglch3@mail2.sysu.edu.cn (L.D.); luoj26@mail.sysu.edu.cn (J.L.); lvyueling88@126.com (Y.L.); 2 Shenzhen Research Institute, Sun Yat-Sen University, Shenzhen 518057, China

**Keywords:** sensorimotor control, visual-motor tracking, MSfApEn, EMG, future information

## Abstract

Visual-motor tracking movement is a common and essential behavior in daily life. However, the contribution of future information to visual-motor tracking performance is not well understood in current research. In this study, the visual-motor tracking performance with and without future-trajectories was compared. Meanwhile, three task demands were designed to investigate their impact. Eighteen healthy young participants were recruited and instructed to track a target on a screen by stretching/flexing their elbow joint. The kinematic signals (elbow joint angle) and surface electromyographic (EMG) signals of biceps and triceps were recorded. The normalized integrated jerk (NIJ) and fuzzy approximate entropy (fApEn) of the joint trajectories, as well as the multiscale fuzzy approximate entropy (MSfApEn) values of the EMG signals, were calculated. Accordingly, the NIJ values with the future-trajectory were significantly lower than those without future-trajectory (*p*-value < 0.01). The smoother movement with future-trajectories might be related to the increasing reliance of feedforward control. When the task demands increased, the fApEn values of joint trajectories increased significantly, as well as the MSfApEn of EMG signals (*p*-value < 0.05). These findings enrich our understanding about visual-motor control with future information.

## 1. Introduction

Sensorimotor control theories hold that the central nervous system (CNS) utilizes expected sensory consequences generated by motor commands for motor planning, and compares with the online sensory feedback originated from specialized senses (vision, audition, vestibular) for real-time monitoring and correction of motor execution [1]. Visual-motor tracking has been widely used to investigate the mechanism of sensorimotor control under various conditions in previous studies. The ever-changing target in visual-motor tracking would reveal more information in sensorimotor control than the stationary target [2,3]. Previous studies mainly focused on the error correction in the process of the motor execution effect through online visual feedback. Bank et al. discovered the effects of visual information (i.e., scaling factor and optical flow density) on motor performance and control strategy during visual tracking tasks [4]. Byblow et al. suggested that central demands increased when attention was directed away from the most relevant visual information, and led to changes in sensorimotor control strategies [5]. Visual-motor tracking training was also considered an effective way to help stroke survivors recover [6]. Therefore, the use of visual-motor tracking is not only beneficial to explore the changes of sensorimotor control, but can also further help solve clinical issues.

The information about future properties showed great potential in improving motor performance. The future visual information about the target can improve the tracking performance by providing knowledge of the future state of the goal [7]. This information was utilized to track the target prospectively, leading to a smaller spatial error, a lower duration, and a better adaption to feedback delays than unpredictable ones during continuous tracking movement [7,8,9]. Furthermore, the longer the duration of information about future behavior, the more motor performance was improved [10]. Apart from the future information, the task demands also have an impact on motor performance. In previous studies, task demands have depended on external force loads, movement speed, and irregularity of target trajectories, etc. [11,12,13]. People respond to changes in task demands by making adjustments to accuracy and amplitude constraints [14,15]. It has been reported that a more difficult task elicited a more vigorous and less smooth movement, which might be the result of less preplanned and more online control than movement in a simple task [16]. Glinka et al. found that participants with higher cognitive and physical demands tended to move less than those with lower demands while standing [17]. In an isometric tracking study, the force output variability increased gradually with the increase in task demands [18]. 

The changes of control strategy in a neuromuscular system can be reflected by the analysis of motor performance. Previous studies analyzed the movement performance of subjects by using the speed and reaction time of simple movement, but these parameters may not be applicable to a more complex trajectory assessment [14,19,20]. Smoothness has been related to velocity, health condition, and even the emotion of participants [13,21,22]. Flash and Hogan found that humans may try their best to control their movements to maximize smoothness under certain conditions [23]. Compared with a series of kinematic analysis methods, normalized integrated jerk (NIJ) was a suitable evaluation of smoothness [24].

Kinematic parameters reflect the external motor performance, which will be influenced by the internal neurological changes [25]. Joint movement is the consequence of the biological forces produced by skeletal muscle contraction. The electromyographic (EMG) signals generated by skeletal muscle contraction contain abundant neuromuscular system information [26]. The internal muscular changes with future information and task demands can be reflected through the analysis of EMG signals. In previous studies, the time and frequency domain methods were commonly used for EMG analysis [27]. The consistency of these methods among multi-measurements needs further improvement as they are easily influenced by EMG amplitude to avoid inconsistent results. [13]. Recent studies have revealed that entropy analysis is a useful method to characterize the complexity of electrophysiological signals and has been widely used in physiological signal analysis [25,28,29,30,31]. Fuzzy approximate entropy (fApEn) can distinguish the complexity of various signals more consistently and have less parameter dependence than Approximate Entropy (ApEn) [28]. Previous studies have shown that fApEn can provide tools to detect the neurological changes by analyzing the complexity of EMG signals [25]. Costa et al. pointed out that the dynamics of a complex nonlinear system are represented on multiple intrinsic scales of the observed time series, and therefore, the entropy estimation calculated on a single scale is not a sufficient descriptor [32]. They proposed using a “coarse-graining” method to extract the various scales of the input data and then calculate entropy estimates for each scale separately, named the multiscale entropy (MSE) method. In recent years, the MSE algorithm has been successfully applied to analyze different kinds of physiological signals [29,33,34]. In this study, the fuzzy approximate entropy after coarse-graining, namely multiscale fuzzy approximate entropy (MSfApEn), was used to analyze the EMG signals in visual motion tracking.

The analysis of joint trajectories and EMG signals comprehensively reflect the influence of task demands and future information on visual motor tracking. Although the effects of future information on visual-motor tracking have been investigated in previous studies, easily predictable target trajectories, such as sinusoids, were applied [9]. The sinusoidal trajectory is easy to be perceived and learned in the tracking tasks, which weakens the effect of future information. The random variation in the target trajectories could avoid the predictability of the target. Target trajectories with different complexity were designed to quantify task demands, including a noise-free sinusoid and sinusoid mixed with random noises. Further, as the task demands also have an important impact on motor performance, three complex levels of target trajectories were designed to explore it. Participants were asked to track the target with or without future-trajectory, respectively. Furthermore, changes in motor performance were assessed by fApEn values and NIJ values of the elbow joint trajectories. The MSfApEn method was used to analyze the complexity of electromyographic signals in biceps and triceps of each time scale.

## 2. Materials and Methods

### 2.1. Participants

First, 18 healthy young adults (9 females, 9 males, mean age of 25.05 ± 1.61) voluntarily participated in this study. All the participants were right-hand dominant and had no known motor and neural impairment history. All the experimental procedures of this study were approved by the ethics committee of Guangdong Work Injury Rehabilitation Center (AF/CS-07/2017.09).

### 2.2. Apparatus and Procedure

**Figure 1:**
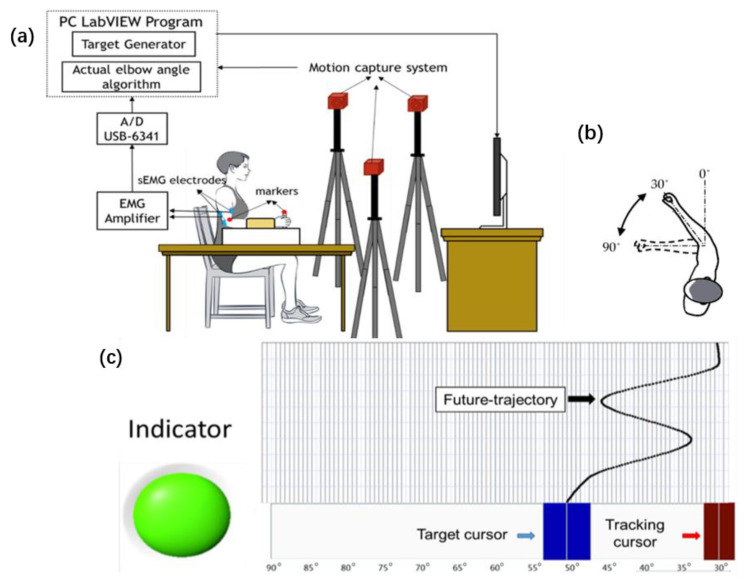
Experimental setup: (**a**) Block diagram of experimental setup; (**b**) diagrammatic representations of the range of the elbow angle; (**c**) interface of tracking tasks on the screen.

Figure 1a,b show the setup of the experiment. Participants were asked to sit in a chair and attach to a handle with their forearm. The handle supported elbow flexion and extension in the horizontal plane. Two circular Ag-AgCl bipolar surface electrodes with a center distance of 2 cm were fixed on the bellies of the biceps and triceps, which provided the main force for flexion and extension of the elbow joint. The surface EMG signals were captured throughout the tracking process by an 8-channel EMG signal acquisition instrument (Shenzhen ThreeG Tech co., Ltd., Shenzhen, China) with a gain of 5000, and then recorded at a 1000 Hz sampling rate by data converters (DAQ-6341, National Instruments, Austin, TX, USA). A motion capture system (OptiTrack, Natural Point, Corvallis, OR, USA) synchronously captured the coordinates of the markers that were attached to the handle and elbow joint at 100 Hz. There was a screen set-up in front of subjects, which provided a real-time display of the target and actual elbow joint angle. The interface displayed on the screen is shown in Figure 1c. The main interface of tracking tasks is shown in Figure 1c. The red and blue rectangles slid left/right along the slide track and was displayed on the screen to provide visual feedback. The blue rectangle (2 cm in width and 4 cm in length) represented the target cursor, the movement trajectory of which was generated by the LabVIEW program. While the red rectangle (1.5 cm in width and 4 cm in length) reflected the real-time elbow angle, the actual elbow joint angle was calculated as the angle between the line formed by the two marks and the extension line of the upper arm. The size of the target cursor was different from that of the control cursor so that subjects could easily distinguish them. There was a module above the slide track that displayed the future-trajectory. In the tasks with future-trajectory, this module displayed the 3 s trajectory of the future in real time. According to previous studies, the 3 s future-trajectories can provide sufficient future visual information for visual-motor tracking [9].

After understanding the experiment protocol, subjects were asked to flex or extend their elbow joints within [30°, 90°] and try their best to catch up with the target cursor. The target cursor moved along 3 kinds of target trajectories. There were 2 kinds of visual conditions designed for each task demand; one of them was set to display a 3 s future-trajectory in a frame above the target cursor and the other not. The experiment involved 6 kinds of combinations corresponding to 3 (target trajectories) × 2 (visual condition). The average fApEn of target trajectories in each combination is shown in Table 1. Participants were asked to perform one combination in each trial, and the duration of each trial was 36 s. Participants were asked to complete 3 blocks after three practice trials. Each block contained 6 different trials described above and the sequence of trials was randomly arranged. Participants were asked to complete all the combinations before moving on to the next block. There was a 5 min break between each block and 30 s interval between each trial.

In order to avoid the adaptation of participants in tracking regular targets, three task demand levels were designed by applying different trajectories during the experiment. The target trajectories were designed as follows [35]:

(1) $$\mathrm{MIX}{\left(P\right)}_{j}=2-Z_{j}X_{j}+X_{j}Y_{j}\;(1<j<N)$$

where *N* is the data length of target trajectories in each trial; $X_{j}=\sqrt{2}\sin(2\pi j/12)$; Y*_j_* is the real random variables with independent identically distributed (i.i.d), which is distributed in the interval [−0.5, 0.5] uniformly. *Z_j_* is an i.i.d random variable composed of 1 and 0, where the probability of 1 is *P* and the probability of 0 is 1 − *P*. The trajectories of the target from levels 1 to 3 were generated by MIX (0), MIX (0.3), and MIX (0.6), respectively. The complexity of target trajectories was quantified by fApEn values. Larger *P* indicates greater demands of the tracking tasks. The target trajectories were created separately, and different noises were mixed with the sinusoid with different subjects. 

### 2.3. Data Analysis

The normalized integrated jerk (NIJ), which removed the influence of tracking duration and distance, was used to measure the movement smoothness. It was calculated as follows [36].

(2) $$NIJ=\sqrt{\frac{duration^{5}}{2\times length^{2}}\times \sum^{\;}jerk{\left(t\right)}^{2}}$$

where *Jerk* is a quantization of the changes in acceleration, and calculated as $\;jerk\left(t\right)=\frac{d^{3}\theta }{dt^{3}}$. The entire joint trajectory was divided into the flexion phase (increase phase of joint angle) and extension phase (decrease phase of joint angle). Both the flexion phase and the extension phase were all calculated in data analysis. The normalized factor *duration^5^*/(2 *× length^2^*) was proposed in the jerk analysis [37] as the duration time in the flexion phase and extension phase was different. The *duration* is the duration time of each phase, and *length* is the data length of each phase. In this study, the sum of all phases was used to evaluate the movement smoothness.

A 20–450 Hz 4th-order Butterworth band-filter was employed to filter the EMG signals. The filtered EMG data from biceps and triceps of 36 s were divided into 6 segments, each of which lasted for 6 s, and the mean value of the 6 segments was calculated as the result of the EMG data. Our previous work combined multiscale entropy with fApEn, and found that the result was susceptible to irrelevant noise by using single-scale entropy [38]. Multiscale fuzzy approximate entropy (MSfApEn), which is a combination of multi-scale entropy and fApEn, was used in this study to reveal the complexity of signals in multiple time scales. The algorithm of MSfApEn consisted of two steps: (1) Coarse-grained process, which was used for information extraction under various time scales; and (2) using fApEn to estimate the complexity of each coarse-grained time series. 

The original signal was set as {*x*(*I*):1 ≤ *I* ≤ *N*}, which is a one-dimensional series. First, the original signal was divided into nonoverlapping segments of length *τ*, called coarse-graining. Second, the coarse-graining signal of each time scale consisted of the average of every segment. The calculation was as follows:

(3) $$y_{j}^{\tau }=1/\tau \sum_{i=\left(j-1\right)\tau +1}^{j\tau }x\left(i\right)\left(1\leq j\leq \frac{N}{\tau }\right)$$

The signal after coarse-graining at time scale 1 (*τ* = 1) was the original signal. The bigger the time scale, the shorter the length(*N*/τ) of the coarse-graining signal. The data length of each trial was 6000, and the time scale *τ* was set from 1 (*N* = 6000) to 20 (*N* = 250). The fApEn was then used to evaluate the complexity of both actual elbow joint angle and coarse-graining EMG signals in each trial [39]:

For a given time series with length *N* {*u*(*i*):1 < *i* < *N*}, dimension *m*, and similar tolerance *r*, the vectors sequence is formed as:

(4) $$X_{i}^{m}=\left\{u\left(i\right),u\left(i+1\right),\ldots ,u\left(i+m-1\right)\right\}-u_{0}\left(i\right)\;\left(i=1,2,\ldots ,N-m+1\right)$$

where $u_{0}=m^{-1}\overset{m-1}{\underset{k=0}{\sum }}u\left(i+k\right)$;

The distance of two vectors, $X_{i}^{m}$ and $Y_{i}^{m}$, is defined as:

(5) $$d_{ij}^{m}=d\left[X_{i}^{m},Y_{i}^{m}\right]=\underset{k\in \left(0,m-1\right)}{\max}\left\{\left|\left[u\left(i+k\right)-u_{0}\left(i\right)\right]-\left[u\left(j+k\right)-u_{0}\left(j\right)\right]\right|\right\}\;\left(i,j=1,2,\ldots ,N-m;\;i\neq j\right)$$

The similarity degree is defined as:

$$D_{ij}^{m}=\mu \left(d_{ij}^{m},n,r\right)$$

where *n* and *r* determine the width of the exponential function and the gradient of the boundary, respectively.

(6) $$f\mathrm{ApEn}\;\left(m,\;r,\;N\right)=\varphi^{m}\left(N,r\right)-\varphi^{m+1}\left(N,r\right)$$

The function $\;\varphi^{m}\left(N,r\right)\;$ was used to average the similarity of each vector in the time series to the others, and the function is defined as:

(7) $$\varphi^{m}\left(N,r\right)=\frac{1}{N-m}\overset{N-m}{\underset{i=1}{\sum }}\left(\frac{1}{N-m-1}\overset{N-m}{\underset{j=1,j\neq i}{\sum }}D_{ij}^{m}\right)$$

Therefore, we could obtain $\varphi^{m+1}\left(N,r\right)$ by

(8) $$\varphi^{m+1}\left(N,r\right)=\frac{1}{N-m-1}\overset{N-m-1}{\underset{i=1}{\sum }}\left(\frac{1}{N-m-2}\overset{N-m-1}{\underset{j=1,j\neq i}{\sum }}D_{ij}^{m+1}\right)$$

Based on previous work, both *m* and *n* were set as 2, and *r* was 0.15 × *std* (signal) in this study.

The multi-scale entropy index (MEI) was used for comparison and analyzed the EMG signals:

(9) $$\mathrm{MEI}=\frac{\sum_{11}^{20}MSfApEn_{\tau }}{10}$$

### 2.4. Statistical Analysis

To judge the effects of future-trajectory and the task demands on the performance of the visual-motor tracking process in this study, a two-factor analysis of variance (repeated-measure ANOVA) was employed. When the significance was obtained, post-hoc pair-t analyses with Bonferroni correction was utilized to identify (1) whether the future information and task requirements had a significant impact on tracking performance; (2) whether there were significant differences in performance between the conditions. The significance level was set at 0.05. SPSS21.0 (SPSS Inc., Chicago, IL, USA) was applied for statistical analysis.

## 3. Result

**Figure 2:**
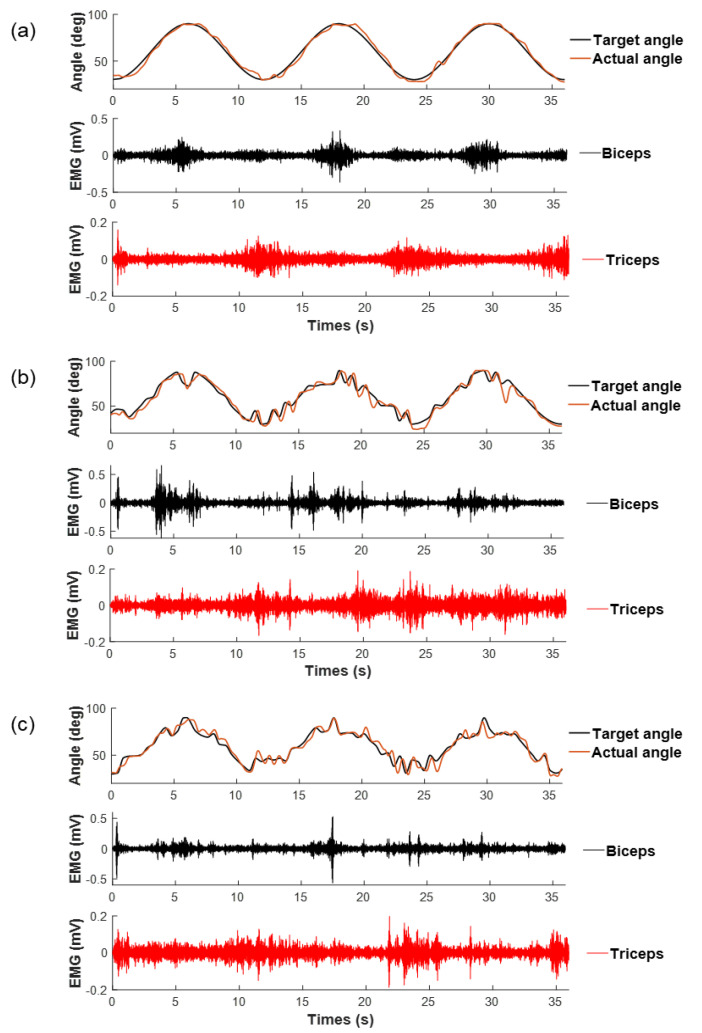
Exemplar of denoised trajectories and electromyographic (EMG) signals of biceps and triceps during tracking tasks in three levels: (**a**) *P* = 0; (**b**) *P* = 0.3; (**c**) *P* = 0.6.

Figure 2 shows an example of target trajectories, actual joint trajectories, and the real-time EMG signals in visual-motor tracking with three different complexities with future-trajectories. The complexity of target and joint trajectories increased with the increase in *p* values. The waveform of the EMG signal of both biceps and triceps changed significantly across different task demand levels.

**Figure 3:**
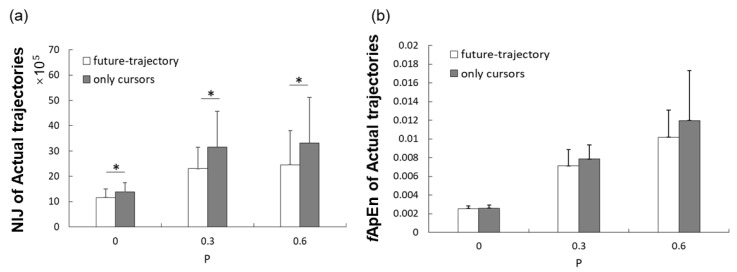
(**a**) Mean and standard deviation (error bars) of the normalized integrated jerk (NIJ) values of actual elbow joint trajectories; (**b**) mean and standard deviation (error bars) of the fApEn values of actual elbow joint trajectories. * Statistically significant difference (*p*-value < 0.05).

The mean NIJ values at three levels with two display modes are shown in Figure 3a. The values of NIJ increased as the target trajectories became more complex. With the displaying of the future-trajectory, subjects traced the target with lower NIJ values than that only showing the current cursor. Two-way ANOVA revealed that there was a significant influence of both task levels (*F* = 22.825, *p*-value < 0.01, *η*^2^ = 0.573) and visual condition (*F* = 6.776, *p*-value = 0.019, *η*^2^ = 0.285) on NIJ values. The paired t-test suggested that tracing for the sine trajectories target had much lower NIJ values than tracing the more complex tasks (*p*-value < 0.001; *p*-value = 0.001). No significant difference was observed between task demand level 2 (*P* = 0.3) and level 3 (*P* = 0.6). The fApEn values of joint trajectories are shown in Figure 3b. The fApEn values of joint trajectories increased as the tasks became more complex, and the mean fApEn value of joint trajectories with future-trajectory was higher than that only showing current cursors. Two-way ANOVA showed that task demands had a significant impact on fApEn values of joint trajectories (*F* = 93.648, *p*-value < 0.001, *η*^2^ = 0.93). However, the fApEn values of joint trajectories with the future-trajectory was not significantly different from those without future-trajectories (*F* = 2.628, *p*-value = 0.087, *η*^2^ = 0.134).

**Figure 4:**
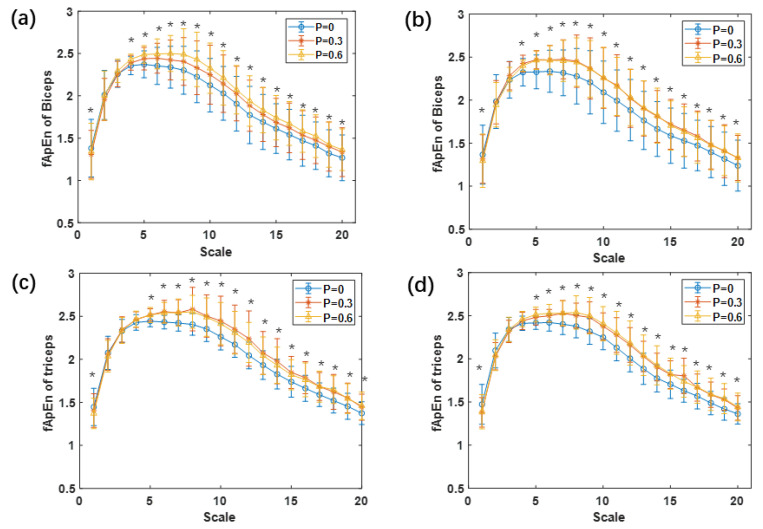
The averaged multiscale fuzzy approximate entropy (MSfApEn) values of all subjects of: (**a**) Biceps EMG signals with future-trajectories; (**b**) biceps EMG signals without future-trajectories; (**c**) triceps EMG signals with future-trajectories; (**d**) triceps EMG signals without future-trajectories. * Statistically significant difference (*p*-value < 0.05).

**Figure 5:**
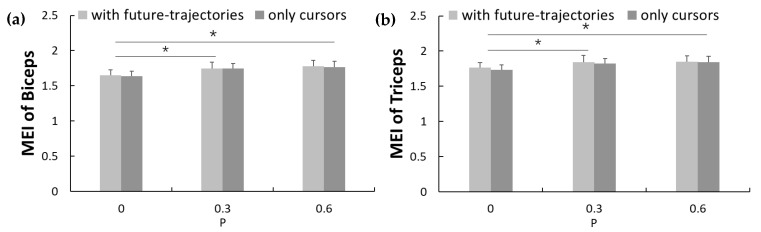
Multi-scale entropy index (MEI) and standard deviation (error bars) of EMG signals: (**a**) Biceps EMG signals; (**b**) triceps EMG signals. * Statistically significant difference (*p*-value < 0.05).

The averaged MSfApEn values of all subjects of biceps and triceps EMG signals are shown in Figure 4. It could be seen from the results that for all visual conditions and task demands, the fuzzy entropy values of both biceps and triceps showed a tendency of first increasing and then decreasing. Specifically, the fuzzy entropy values increased significantly on the scale of 1–5, and then decreased gradually on the scale of 5–20 to the level equivalent to the scale of 1. The results of two-way ANOVA analysis revealed that the time scale factor significantly influenced the fuzzy entropy values of the EMG of the biceps (*F* = 37.870, *p*-value < 0.001, *η*^2^ = 0.666) and triceps *(F* = 47.725, *p*-value < 0.001, *η*^2^ = 0.715). In addition, task demands also affected the EMG of biceps (*F* = 55.774, *p*-value < 0.001, *η*^2^ = 0.746) and triceps (*F* = 38.084, *p*-value < 0.001, *η*^2^ = 0.667). Especially when the time scale factor was 5–20, the fuzzy entropy values of biceps and triceps EMG signals showed significant differences under different task demands (all *p*-values < 0.05). The mean *MEI* of both biceps and triceps as shown in Figure 5 was significant lower for fewer task demands than higher ones (*F* = 21.730, *p*-values < 0.001, *η*^2^ = 0.626; *F* = 10.949, *p*-values = 0.002, *η*^2^ = 0.457). The paired t-test revealed that the *MEI* of level 2 and level 3 were significantly lower than that of level 1(*P* = 0) with and without future-trajectories of targets (*p*-values < 0.05), but there was no significant difference between that of level 2 and level 3. The *MEI* of both biceps and triceps EMG slightly reduced but showed no significant difference between the tracking tasks with and without future-trajectories (*F* = 0.635, *p*-values = 0.44, *η*^2^ = 0.047; *F* = 3.269, *p*-values = 0.094, *η*^2^ = 0.201).

## 4. Discussion

This study devoted to exploring the changes in motor performance and EMG signals among healthy adults with and without future-trajectory, and the target trajectories were mixed with random noises to increase the unpredictability of the target. The NIJ values of real tracking trajectories reflected the smoothness of the elbow joint, and the complex variations in joint trajectories and neuromuscular control were represented by fApEn values of elbow joint trajectories and EMG signals, respectively. In particular, our study quantifies the regularity of EMG signals on different time scales by coarse-grained processing of the original EMG signals, to improve the accuracy of entropy estimation.

### 4.1. Effects of Future Information on the Joint Trajectories and EMG Signals 

The lower NIJ values suggested a smoother elbow flexion-extension joint trajectory with future-trajectory than without it. The changes in movement smoothness may be the consequence of the different control strategies in motor execution when the task demands were the same [40]. Previous studies suggested that sensorimotor control was based on the optimal integration of sensory feedback with our predictions, and tended to minimize the adverse effects of noises to achieve more stable and accurate motor control [41,42]. In this integration, more reliable information sources should gain a higher weight [43]. Displaying the future-trajectory in tracking tasks may increase the reliability of the prediction, resulting in an increased weight of the feedforward control, which is an open loop control with negligible delay and has a lower degree of noise than the feedback control. The application of the target’s future-trajectory might facilitate the generation of more accurate predictions in the forward model [44]. With these predictions, participants were able to catch the target and reduce sub-movements during the tracking process for error correction [45], and smooth the trajectories of joints. In this study, the tasks with future-trajectories incite feedforward control to a greater extent in sensorimotor control than those without future-trajectories, and resulted in a significant decrease in NIJ values. Norio et al. also found the influence of visual condition on the smoothness of the upper limb movement [46]. 

Through the calculation of multi-scale fApEn, the fApEn values of EMG signals were increased from scale 1 to 5, but decreased continuously from scale 5 to 20, which indicated that the time scale had an effect on the analysis of physiological signals. As the surface EMG signal was easily affected by nonphysiological and physiological factors, the collected EMG signals in our study might be confused with irrelevant noises. According to a study on gait leg muscles during treadmill walking, both the old and the young participants showed a low entropy value at larger scales [47], which suggested that entropy analysis of a large time scale could eliminate the influence of irrelevant noise. In addition, previous studies have also demonstrated that the coarse-grained process can gradually filter out random components that are unrelated, such as white noise [32]. The rise at small scales and the fall at large scales might be influenced by the time scale and noises. As shown in Figure 3, the slightly reduced fApEn values of elbow joint trajectories indicated fewer complex movements with the future-trajectory. The fApEn values of the EMG of triceps increased significantly with future-trajectory, which could be influenced by the number of recruited motor fibers, firing rate, modification of motor fiber conduction velocity, and motor unit synchronization [48].

### 4.2. Effects of Task Demands on the Joint Trajectories and EMG Signals

The rise in MEI caused by the increasing task demands represent the irregular EMG signals of both biceps and triceps, which indicated less predictable muscle firing patterns [49]. Previous studies found that the task demands influenced the muscle patterns through the number of recruited motor fibers and firing rates of motor units [50,51]. In addition, Enders et al. pointed out that increased task demands altered the neuromuscular control strategy and resulted in an increased structure in motor execution [52], which also led to more unpredictable muscle firing patterns. However, Murrilo et al. observed an inverse relationship between the complexity of movement and EMG signals when exploring the effects of task difficulty on posture sway [53]. As the smaller time scales fApEn were susceptible to irrelevant noise [38], we speculated that the conflicting results might be caused by the noises when using the single-scale entropy analysis in this study. With the increase in task demands, both the complexity of joint trajectories and movement smoothness increased significantly. The task demands of each trial were quantified by fApEn values of the target trajectory. A previous study also reported that subjects adopt different strategies to control their movements when task demands changed [54]. A point-to-point aiming experiment of 30 participants also showed the same results that when the task difficulty increased, both the young and the old would generate less smooth movement [14]. Ma’s study also suggested that a more complex task elicited less smooth movements in the elder subjects [16].

The limitation of this study was that only three kinds of task demands were set in this experiment. Further study could refine the task requirements to explore more subtle changes in joint trajectories and EMG signals. Another limitation is that we deduced that the smoothness of joint movement might be the result of the increased weight of feedforward control in sensorimotor control from the analysis of experimental results when discussing the influence of future information on joint trajectories during visual-motor tracking. However, further neuromuscular system studies are needed to verify the accuracy of this inference.

## 5. Conclusions

This study investigated the joint trajectories and EMG signals responses of healthy adults with and without future-trajectories in different task demands. The results indicated that the smoothness of visual-motor tracking was significantly affected by task demands and future information of the target, which might be the result of the increasing weight of feedforward control in sensorimotor control strategies. The complexity of biceps and triceps EMG signals, as well as elbow joint trajectories, was mainly affected by task demand, resulting in less predictable muscle firing patterns. These findings can enrich our understanding of the visual-motor control, as well as provide a basis for the design of rehabilitation training tasks for movement disorders in clinical practice.

## Figures and Tables

**Table 1:** Task conditions and the average fuzzy approximate entropy (fApEn) of target trajectories in each condition.

Task Demand	*P*	Visual Condition	fApEn of Target Trajectories	
			Mean	SD
Level 1	0	with future-trajectory	0.00183	3.07 × 10^−8^
		without future-trajectory	0.00183	2.27 × 10^−8^
Level 2	0.3	with future-trajectory	0.00681	0.00034
		without future-trajectory	0.00724	0.00048
Level 3	0.6	with future-trajectory	0.01090	0.00068
		without future-trajectory	0.01083	0.00066

## Data Availability

The data presented in this study are available on request from the corresponding author.
